# FOXF1 Mediates Endothelial Progenitor Functions and Regulates Vascular Sprouting

**DOI:** 10.3389/fbioe.2018.00076

**Published:** 2018-06-14

**Authors:** Caterina Sturtzel, Karoline Lipnik, Renate Hofer-Warbinek, Julia Testori, Bettina Ebner, Jaqueline Seigner, Ping Qiu, Martin Bilban, Anita Jandrositz, Karl-Heinz Preisegger, Gerold Untergasser, Eberhard Gunsilius, Rainer de Martin, Jens Kroll, Erhard Hofer

**Affiliations:** ^1^Department of Vascular Biology and Thrombosis Research, Center for Physiology and Pharmacology, Medical University of Vienna, Vienna, Austria; ^2^Department of Laboratory Medicine & Core Facility Genomics, Core Facilities, Medical University of Vienna, Vienna, Austria; ^3^VivoCell Biosolutions GmbH, Graz, Austria; ^4^Institut für morphologische Analytik und Humangenetik, Graz, Austria; ^5^Laboratory for Tumor Biology & Angiogenesis, Medical University of Innsbruck, Innsbruck, Austria; ^6^Department of Vascular Biology and Tumor Angiogenesis, European for Center for Angioscience, Medical Faculty Mannheim of Heidelberg University, Mannheim, Germany

**Keywords:** endothelial progenitors, ECFC, vascular sprouting, FOXF1, Notch2, ephrinB2, intersegmental capillaries

## Abstract

Endothelial colony forming cells (ECFC) or late blood outgrowth endothelial cells (BOEC) have been proposed to contribute to neovascularization in humans. Exploring genes characteristic for the progenitor status of ECFC we have identified the forkhead box transcription factor FOXF1 to be selectively expressed in ECFC compared to mature endothelial cells isolated from the vessel wall. Analyzing the role of FOXF1 by gain- and loss-of-function studies we detected a strong impact of FOXF1 expression on the particularly high sprouting capabilities of endothelial progenitors. This apparently relates to the regulation of expression of several surface receptors. First, FOXF1 overexpression specifically induces the expression of Notch2 receptors and induces sprouting. Vice versa, knock-down of FOXF1 and Notch2 reduces sprouting. In addition, FOXF1 augments the expression of VEGF receptor-2 and of the arterial marker ephrin B2, whereas it downmodulates the venous marker EphB4. In line with these findings on human endothelial progenitors, we further show that knockdown of FOXF1 in the zebrafish model alters, during embryonic development, the regular formation of vasculature by sprouting. Hence, these findings support a crucial role of FOXF1 for endothelial progenitors and connected vascular sprouting as it may be relevant for tissue neovascularization. It further implicates Notch2, VEGF receptor-2, and ephrin B2 as downstream mediators of FOXF1 functions.

## Introduction

Endothelial colony forming cells (ECFC), also termed blood outgrowth endothelial cells (BOEC), can be easily outgrown from human cord blood or adult peripheral blood using standard endothelial cell growth conditions (Yoder et al., 2007; Martin-Ramirez et al., 2012; Hofer-Warbinek et al., 2014; Medina et al., 2017). Due to their high proliferative potential they can be obtained in large quantities suitable for regenerative medicine. Progenitor cells comparable to ECFC can also be generated from induced pluripotent stem cells (Prasain et al., 2014). In distinction from other so-called circulating endothelial progenitor cells (EPC), that can be obtained from blood by different isolation and culture conditions and are of hematopoietic origin, ECFC have characteristics of true endothelial progenitors. They form vascular networks and integrate into newly formed vessels *in vivo* (Dubois et al., 2010; Banno and Yoder, 2017). It has been proposed that ECFC originate from the endothelial lining of blood vessels and lung capillaries could be a major source for their shedding into the bloodstream (Alphonse et al., 2014, 2015). ECFC could therefore constitute a vascular organ-specific progenitor cell type involved in regeneration of endothelium and neovascularization. Although ECFC are difficult to obtain from murine blood, an endothelial progenitor/stem-like cell population has been also located at the inner surface of murine blood vessels (Naito et al., 2012).

Irrespective of their still debated origin and normal physiological role, ECFC are promising candidates for cell therapies. When transplanted into sites of ischemic injury, ECFC incorporate into damaged blood vessels improving blood perfusion and supporting repair processes and organ function (Schwarz et al., 2012; Alphonse et al., 2014; Palii et al., 2014; Banno and Yoder, 2017). Based on their simple isolation (Martin-Ramirez et al., 2012; Alphonse et al., 2015) and properties they are also primary candidates for the *ex vivo* generation of vascularized tissue batches or organs for regenerative therapies (Ruvinov and Cohen, 2014).

Given their therapeutic importance, a precise characterization of the cells in comparison to mature endothelial cells of the vessel wall is needed. In regard of relevant surface markers ECFC were reported to be indistinguishable from mature endothelial cells, but ECFC are distinct by their clonal growth properties and high proliferative capacity (Banno and Yoder, 2017). To further characterize ECFC we have undertaken transcriptional profiling and have identified FOXF1 to be the most preferentially expressed transcription factor in ECFC when compared to mature endothelial cells.

The FOX (forkhead box) family of transcription factors is generally involved in the determination of cell lineage and organ specificities. For example, FOXA is a pioneer transcription factor regulating accessible nucleosome configurations at enhancers for liver-specific genes (Iwafuchi-Doi et al., 2016), FOXP1 promotes neural stem cell differentiation (Braccioli et al., 2017) and FOXN1 has been used in the reprograming of fibroblasts for the formation of an ectopic thymus (Bredenkamp et al., 2014). In the vascular system it has been shown that FOXC factors are required for vascular development (Seo et al., 2006; De Val et al., 2008) and more recently that FOXF1 is involved in formation of embryonic vasculature by regulating VEGF receptor genes (Ren et al., 2014).

Whereas initial vessel formation in the embryo occurs via vasculogenesis, the assembly of angioblasts, most vessel formation during growth and in the adult is initiated by vascular sprouting, i.e., angiogenesis (Risau, 1997). In this process an endothelial tip cell, starting from an existing vessel, invades into the surrounding tissue. Tip cells follow an increasing gradient of VEGF-A generated by ischemic tissues that they sense via VEGF receptor-2 and in part also VEGF receptor-3 (Adams and Alitalo, 2007; Blanco and Gerhardt, 2013). The growing vascular sprout is further formed by so-called endothelial stalk cells that follow the tip cell and have the capacity to proliferate leading to sprout extension. Sprout growth and interplay between tip and stalk cells is regulated by an intricate balance of signals involving, aside the attracting VEGF-A/VEGFR2 and − 3 interactions, also repelling guidance cues (Adams and Eichmann, 2010) and Notch receptors and their ligands (Ehling et al., 2013). For example, the Notch ligand Dll4 expressed on tip cells interacts with Notch1 receptors on stalk cells to prevent additional tip cell formation.

Specification and separation into arterial or venous capillaries and vessels further involves signaling mediated via Ephrin receptors and their ligands (Sawamiphak et al., 2010; Salvucci and Tosato, 2012). Whereas ephrin B2 characterizes arterial endothelial cells, EphB4 receptors are preferentially expressed on venous endothelial cells.

An excellent model to investigate vessel formation and its modulation is the zebrafish (Hogan and Schulte-Merker, 2017). In the still transparent larvae at 23 to 48 h post fertilization the intersegmental arterial vessels are formed by sprouting from the dorsal artery. Processes involving arterial sprouting can therefore be easily analyzed by live imaging during this time period. In addition, formation of the venous and lymphatic systems can be analyzed (Isogai et al., 2001).

Here we report that FOXF1 is selectively expressed in ECFC and that FOXF1 expression is linked to the high sprouting and tip cell formation capacities of ECFC. Furthermore, we determine that FOXF1 expression can modulate Notch2 receptors and that reducing Notch2 expression inhibits sprouting comparable to downmodulation of FOXF1. In addition, FOXF1 augments VEGFR-2 expression and seems to preferentially support arterial vessel sprouting as it upregulates ephrinB2 and downregulates EphB4. A role of FOXF1 for arterial vessel formation is also corroborated by the finding that downmodulation of FOXF1 in zebrafish deteriorates arterial vessel formation by sprouting. Taken together the data support that FOXF1 is an important determiner of the progenitor status of ECFC and regulates sprouting capabilities.

## Materials and methods

### Cell culture

Endothelial colony forming cells (ECFCs) were isolated from human cord blood samples obtained from the Department of Obstetrics and Gynecology, Medical University of Vienna, or supplied by VivoCell AG (Graz, Austria). Procedures of cord blood collection including a written informed consent have been approved for this study by the ethical committee of the Medical University of Vienna (protocol number 122/2010). Blood samples were collected in cord blood collection bags (MacoPharma, Mouvaux, France) and stored at room temperature. Then mononuclear cells (MNC) were isolated within 24 h over Lymphocyte Separation Medium LSM 1077 (PAA, now GE Healthcare) as recommended by the manufacturer. ECFC were obtained from the MNCs according to published procedures (Yoder et al., 2007; Lucas et al., 2009). In short, MNC were resuspended in microvascular endothelial cell growth medium-2 (EGM-2 MV medium, Bio Whittacker, Lonza) and seeded onto 0.01% kangaroo or 0.1% rat collagen (Sigma-Aldrich) coated culture dishes. Twenty four hours after seeding the floating cells were removed and fresh medium was added to the adherent cells. Clonal outgrowth was observed after 2–6 weeks. ECFCs were expanded on 1% gelatin coated cell culture dishes in EGM-2 MV medium and used for further analysis.

Human umbilical vein endothelial cells (HUVECs) were isolated as described previously (Testori et al., 2011) and cultured on 1% gelatin coated cell culture dishes in EGM-2 MV medium. HUVECs of passage 2–3 were used for experiments.

Human embryonic kidney 293 cells (HEK 293 cells, ATCC No. CRL-1573) were cultured in Minimum Essential Medium α (MEM-α, Invitrogen LT, Carlsbad, CA) supplemented with 10% neonatal calf serum (NCS, Invitrogen LT), 2 mM glutamine, 100 U/ml penicillin and 1 mg/ml streptomycin (all PAA/now part of GE).

HEK 293T/17 cells (ATCC No. CRL-11268) were cultured in Dulbecco's Modified Eagle's Medium (DMEM, Invitrogen LT) with 10% fetal calf serum (FCS, Sigma-Aldrich), 2 mM glutamine, 100 U/ml penicillin and 1 mg/ml streptomycin.

All cells were maintained at 37°C applying 5% CO_2_ and 95% humidity.

### Immunocytochemistry

For immunostaining cells were seeded in chamber slides (Lab-Tek Brand Products; Nalge Nunc International). The day after seeding cells were fixed with 4% paraformaldehyde (PFA) at room temperature for 10 min. The murine anti-human CD31-FITC (R&D Systems), polyclonal anti-VE-cadherin-FITC (Bender MedSystems GmbH) as well as the sheep anti-human vWF-FITC (AbD Serotec) were diluted 1:100 in 1% BSA-PBS and incubated at 4°C overnight. For nuclear staining, cells were incubated with 1 ng/ml Hoechst stain (Sigma) for 5 min. Thereafter immunofluorescence was analyzed with a fluorescence microscope (Nikon eclipse 80i). Images were taken with an integrated CCD camera (Nikon) at indicated magnifications.

### Flow cytometry

For flow cytometry cells were harvested by treatment with accutase (PAA, Pasching, Austria). Cells were stained for VEGFR-2 in PBS containing 0.5% BSA and 2 mM EDTA with murine anti-human KDR-APC (R&D Systems) antibodies as recommended by the manufacturer. As isotype controls, IgG1-APC antibodies (Miltenyi) were used. Antibody binding was assessed on a FACSCalibur and evaluated using CellQuest software (BD Biosciences. San Jose, CA).

### RNA preparation, RT-PCR and real-time RT-PCR analysis

For the extraction of RNA, cells were incubated with RNAlater (Ambion) for 1 min, washed with water and lysed in QIAzol (Qiagen). RNA was extracted according to manufacturer's instructions. 1 μg of total RNA was used to synthesize cDNA with Superscript II Reverse Transcriptase (Invitrogen LT) and oligo(dT) primers (Invitrogen LT) according to the recommended protocol.

Semi-quantitative RT-PCR was performed with GoTaq^TM^ DNA polymerase (Promega) according to the instructions of the producer. For quantitative real-time RT-PCR a Rotor-Gene®Q LightCycler (Qiagen) detecting Rotor Gene SYBR Green (Qiagen) was used. All values were normalized to β-actin mRNA as internal standard. Oligonucleotide primers and annealing temperatures used for gene amplification are listed in Supplementary Table S2.

### Affymetrix microarray hybridization

Extracted RNA was further purified using RNeasy kit (Qiagen). Total RNA (200 ng) was analyzed on genome-wide human Gene Level 1.0 ST GeneChips (Affymetrix, Santa Clara, CA, USA) as described in detail in Tauber et al. (2010). Scanning of the arrays was carried out according to manufacturer's protocols https://www.affymetrix.com. RMA signal extraction, normalization, and filtering was performed as described (http://www.bioconductor.org/). A variation filter was applied for selecting informative (i.e., significantly varying) genes. The filtering criteria for the exemplary data sets required an interquartile range > 0.5 and at least one sample with expression intensity > 50. The full obtained data sets are now available at Gene Expression Omnibus under the accession number GSE22695 (http://www.ncbi.nlm.nih.gov/geo/query/acc.cgi?acc=GSE22695).

### Generation of replication-defective adenoviruses encoding FOXF1

A full-length open reading frame cDNA clone (BC089442) encoding for the human FOXF1 gene was ordered from ImaGenes (Berlin, Germany). The complete coding region was amplified from the cDNA clone described above using PCR primers containing additionally designed SalI (5′-primer) or XhoI (3′-primer) restriction sites and inserted into the respective restriction enzyme cleavage sites of the multiple cloning site of the pShuttle-IRES-hrGFP-1 vector plasmid, which is part of the AdEasyTM Adenoviral Vector System kit from Stratagene (Agilent Genomics, La Jolla, CA). pShuttle.FOXF1 and the pAdEasy-1 were co-transformed into supplied BJ5183 RecA^+^ E. coli for recombination. The successfully recombined vector was sequenced for accuracy, linearized with PacI (New England Biolabs) and transfected into HEK293 cells using a mammalian transfection kit (Stratagene) to generate primary adenoviruses. These were subcloned, amplified and purified by ultracentrifugation over a CsCl_2_ gradient (Sigma-Aldrich) as previously described (Testori et al., 2011). An empty control adenovirus was prepared in parallel. Viral titer was determined using the Adeno-X rapid titer kit (Clontech, Mountain View, CA) according to the recommended protocol.

### Generation of replication defective lentiviruses

Lentiviral vector DNA encoding shRNA directed against FOXF1 (pLKO.1-shRNA-FOXF1-puro), shRNA directed against Notch2 (pLKO.1-shRNA-Notch-2-puro) or scrambled shRNA (pLKO.1-shRNA-neg-puro) were obtained from Sigma-Aldrich. The packaging plasmids psPAX2 and pMD2.G were from Addgene (Cambridge, MA, USA). For the generation of lentiviral vehicles the lentivirus vector plasmids were co-transfected with the packaging plasmids into HEK 293T/17 cells using an optimized calcium phosphate method (Stratagene, LaJolla, CA) according to the recommendations of the manufacturer. Eight hours after transfection medium was changed and then viral supernatant was harvested 24 and 48 h later. For infection the 0.4 μm filtered virus supernatants were added to subconfluent ECFCs together with fresh medium (EGM2-MV) in a ratio of 1:1.

### SDS-page and western blot analysis

Equal amounts of protein extracts from cells were separated by 10% SDS-polyacrylamide gel electrophoresis (PAGE) and transferred onto an Immobilon-P membrane (Millipore, Bedford, MA, USA) by semidry blotting. Membranes were blocked for 1 h in 5% organic skimmed milk (Sigma-Aldrich) in PBS containing 0.1% Tween-20 (Bio-Rad). For protein detection membranes were incubated in PBS/0.1% Tween-20/5% milk powder containing a 1:1000 diluted goat polyclonal anti-hFOXF1 antibody (R&D Systems) for endogenous FOXF1 expression or a 1:5000 dilution of the antibody for the visualization of the adenovirus mediated FOXF1 expression. The rat monoclonal anti-hNotch2 intracellular domain antibody (R&D systems) was used at a dilution of 1:500. Bound antibodies were detected by applying species-specific HRP-conjugated secondary antibody (GE Healthcare, city, state; diluted 1:5000) followed by enhanced chemiluminescence (GE Healthcare). As an internal control glyceraldehyde-3-phosphate dehydrogenase (GAPDH) was detected using a monoclonal mouse anti-GAPDH antibody (Millipore-Chemicon International, Vienna, Austria; diluted 1:5000).

### Spheroid-based *in vitro* sprouting assay

ECFCs transduced with adeno- or lentiviruses were tested for their ability of sprout formation into an extracellular matrix similar setting upon stimulation based on the protocol of Korff and Augustin (1998) as described in Testori et al. (2011). Briefly, ECFC were suspended in culture medium containing 20% (wt/vol) methylcellulose (Sigma-Aldrich) to generate spheroids of defined cell number (400 cells/spheroid) in hanging drops overnight. In PBS/10% FCS harvested spheroids were embedded into rat collagen gels with the final concentration of 40% Methocel/10% FCS/ 40% collagen/10% 10xM199 (Sigma-Aldrich) and transferred into 24-well plates for suspension cells. After 30 min of gel formation at 37°C, EBM-2 MV without or containing VEGF and/or bFGF (50 ng/ml each) was layered onto the top of the gel. Following incubation at 37°C overnight, *in vitro* angiogenesis was stopped by fixation using 10 % PFA for 1 h. Pictures were taken on a Nikon eclipse 80i microscope equipped with a CCD camera (Nikon). Accumulated sprout length of at least 20 spheroids per condition were measured with ImageJ software. All experiments were performed at least three times with ECFCs of different donors.

### Knock-down of FOXF1 in zebrafish

Embryos of the Tg(fli1:EGFP)y1 line were raised and staged as recently described (She et al., 2018). Embryos were kept in E3 medium (5 mM NaCl, 0.17 mM KCl, 0.33 mM CaCl2, 5–10% methylene blue) at 28.5°C with or without 0.003% 1-phenyl-2-thiourea (Sigma) to suppress pigmentation and staged according to somite number or hours post-fertilization (hpf). All experimental procedures on animals were approved by the local government authority, Regierungspräsidium Karlsruhe (license no.: 35-9185.64) and carried out in accordance with the approved guidelines.

Morpholinos (Gene Tools) were diluted in 0.1 M KCl, used by the indicated concentrations and injected through the chorion of one-cell or two-cell stage embryos. Splice blocking morpholino FoxF1 SB-FoxF1-MO (5′-CTTAAAAACTTTACCTTGGAGGTCG−3′) or a standard control morpholino (5′-CCTCTTACCTCAGTTACAATTTATA-3′) together with a p53 morpholino (Epting et al., 2010) were used and dose escalation studies were performed to determine submaximal morpholino concentrations. Knockdown efficiency was confirmed by RT-PCR with the primers 5′-GCCCCCGACATCTCTAAATA-3′ and 5′-TGTCACACATGCTGGGAGAT-3′ for FoxF1. PCR was conducted at 95°C for 5 min, (95°C for 30s, 56°C for 30s, 72°C for 45s) × 35 cycles and 72°C for 5 min and PCR products were loaded on a agarose gel. Tg(fli1:EGFP)y1 embryos were manually dechorionated and anesthetized with 0.05 % tricaine (Sigma). Morphological analysis of vessels was performed using a CTR 6000 microscope (Leica) or a TCS SP5 system (Leica). For quantitative morphological analysis the number of embryos with non-defective, partially defective and defective intersegmental vessels (ISVs), dorsal longitudinal anastomotic vessel (DLAV) connections between the investigated ISVs in the trunk vasculature, parachordal lymphangioblast (PLs), and thoracic duct (TD) was determined at 48 or 120 hpf, respectively. Results are given in percentage.

### Statistics

Statistical analysis was performed with Prism 6 software (GraphPad, San Diego, CA, USA) using paired Student's *t*-test. A *p*-value of 0.05 was considered as statistically significant.

## Results

### FOXF1 is preferentially expressed in blood ECFC when compared to vessel wall endothelial cells

To decipher genes specifically expressed in endothelial progenitors of the ECFC type we have comparatively analyzed gene expression profiles of ECFC isolated from blood and of terminally differentiated endothelial cells of the vessel wall. For this purpose, we isolated ECFC from human cord blood or adult peripheral blood and mature endothelial cells from human umbilical cords or adult veins. Usually about 5 to 10 ECFC colonies were obtained from mononuclear cells of 50 ml of cord blood or adult peripheral blood. For comparative reasons, to display a distinct gene expression profile to hematopoietic progenitor cells, we also isolated CD34 positive cells from cord blood.

The transcriptomic profiles of low passage ECFC from cord blood were evaluated in comparison to HUVEC and of ECFC from adult peripheral blood in comparison to adult saphenous vein endothelial cells (HSVEC). When focusing our analysis on transcriptional regulators, we found two transcription factors more than 5-fold overrepresented in the ECFC of both sources, the winged helix transcription factor FOXF1 and the Krueppel-related zinc finger protein 117 (Supplemental Table S1). FOXF1 was the most preferentially expressed transcription factor in both ECFC (10–20-fold). In contrast, its expression was below background levels in CD34^+^ cells from cord blood supporting an endothelial progenitor and non-hematopoietic progenitor function. In addition, the data obtained provide evidence that otherwise the ECFC used in this study are highly similar to mature endothelial cells in the expression of endothelial markers such as VEGFR-2, Tie-2, VE-cadherin, and von Willebrand factor (Supplementary Table S2). Since ECFC from cord blood were more easily obtained and seemed to have advantageous proliferation potential we focused further work on the role of FOXF1 in cord blood-derived ECFC.

First we reassessed the transcriptomic profiling data by investigating the mRNA levels of FOXF1 by realtime RT-PCR as well as by Western Blot analysis using multiple isolates of ECFC (*n* = 12) and HUVEC (*n* = 11) derived from different donors at passage 2–3. In parallel, the expanded cells were analyzed for markers of endothelial and hematopoietic cells by immunocytochemistry.

**Figure 1:**
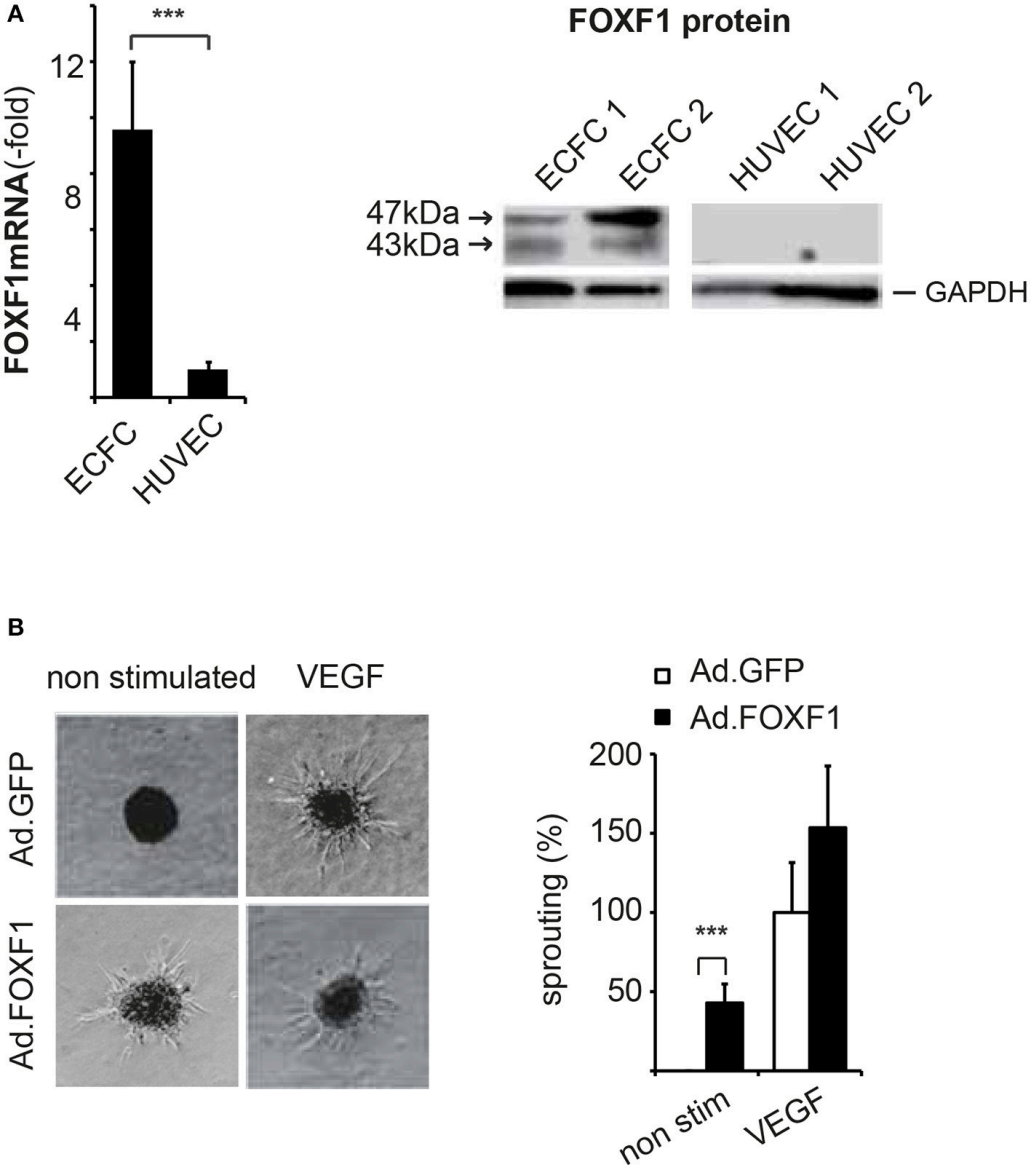
FOXF1 is preferentially expressed in ECFC and induces sprouting. **(A)** Real-time RT-PCR and Western blot analysis of FOXF1 mRNA and protein in ECFC and HUVEC: ECFC and HUVEC were isolated from cord blood and umbilical cords, respectively, and cultured to density in 6-well plates as described in the Methods section. First to second passage cells were used for mRNA isolation, cDNA syntheses, and real-time RT-PCR or were lysed in sample buffer, the proteins separated by polyacrylamide gel electrophoresis and Western blotted. The left part shows the overrepresentation of FOXF1 mRNA in samples from ECFC compared to those of HUVEC. Mean values ± SEM of at least 10 different donors are shown, asterisks indicate statistical significance of difference. The right part shows the selective expression of FOXF1 protein in Western blots of ECFC compared to HUVEC. Two different isolates of ECFC and HUVEC representative of 10 analyzed are shown. Membranes were probed for FOXF1 and GAPDH as internal standard using corresponding antibodies. **(B)** Overexpression of FOXF1 induces sprouting in the spheroid assay: ECFC were infected with Ad.FOXF1 or Ad.GFP with an MOI of 8. The following day spheroids were generated and embedded into collagen gels without or in the presence of VEGF (100 ng/mL) as described in the Methods section. After 24 hours spheroids were fixed and photographic images taken. Representative images of spheroids generated from Ad.FOXF1- or Ad.GFP-infected cells are displayed in the left panel. The quantification of the cumulative sprout length is depicted in the right panel. Results are displayed as mean values ± SEM. The cumulative sprout lengths observed for spheroids transduced with control adenoviruses (Ad.GFP) and induced with VEGF were arbitrarily set to 100%. One representative experiment of four performed is shown (^***^p < 0.001).

Consistent with the microarray analysis FOXF1 mRNA was on average 10-fold higher in ECFC when compared to HUVEC and FOXF1 protein was strongly stained in Western blots of ECFC lysates, whereas it was undetectable in HUVEC lysates (Figure 1A). ECFC regularly displayed strong staining for surface markers of the endothelial cell lineage such as CD31, VE-cadherin, and vWF (Supplementary Figure S1). Other markers of hematopoietic cells tested such as CD45 and CD14 could never be detected verifying that the isolated ECFC were not significantly contaminated with other cell types of hematopoietic origin (data not shown). This is also supported by the absence of signals for CD45, CD14, and CD133 in the microarray data of ECFC, whereas strong expression of these genes is readily detected in blood CD34^+^ cells (Supplementary Figure S2).

### FOXF1 controls a program that provides increased sprouting capacity to ECFC

Despite displaying surface markers very similar to mature endothelial cells, ECFC are diverse by their potential to proliferate over extended time periods (Yoder et al., 2007) and to integrate into newly forming blood vessel *in vivo* (Dubois et al., 2010). We have regularly found that the capacity of ECFC to form tubular sprouts *in vitro* is significantly higher than for HUVEC (Schneller and Hofer, unpublished observation).

To show a potential involvement of FOXF1 in this property we performed gain- and loss-of-function studies. For this purpose, we produced on the one hand a replication defective adenoviral vector encoding FOXF1, which mediates strong FOXF1 overexpression as confirmed by realtime RT-PCR and Western blot analysis (Supplementary Figure S2A). On the other hand, we used lentiviruses expressing short hairpin RNAs (shRNAs) to downregulate endogenous FOXF1 expression levels. Up to 90% decrease in FOXF1 mRNA expression was achieved 48 h after infection leading to a strong reduction of FOXF1 protein (Supplementary Figure S2B).

Indeed, when we analyzed the cumulative lengths of sprouts formed by ECFC without and in the presence of adenoviral overexpression of FOXF1 a strong induction of basal sprouting activity without addition of proangiogenic factors was revealed (Figure 1B). When VEGF-A-induced sprouting was analyzed, no statistically significant increase for FOXF1 overexpressing cells was observed, although the data seemed to display a tendency for increased sprouting also in this case. Much more prominent, however, was the FOXF1-elicited sprouting in the absence of exogenous angiogenic factors which resulted in nearly 50% of the cumulative sprout length triggered by VEGF-A.

**Figure 2:**
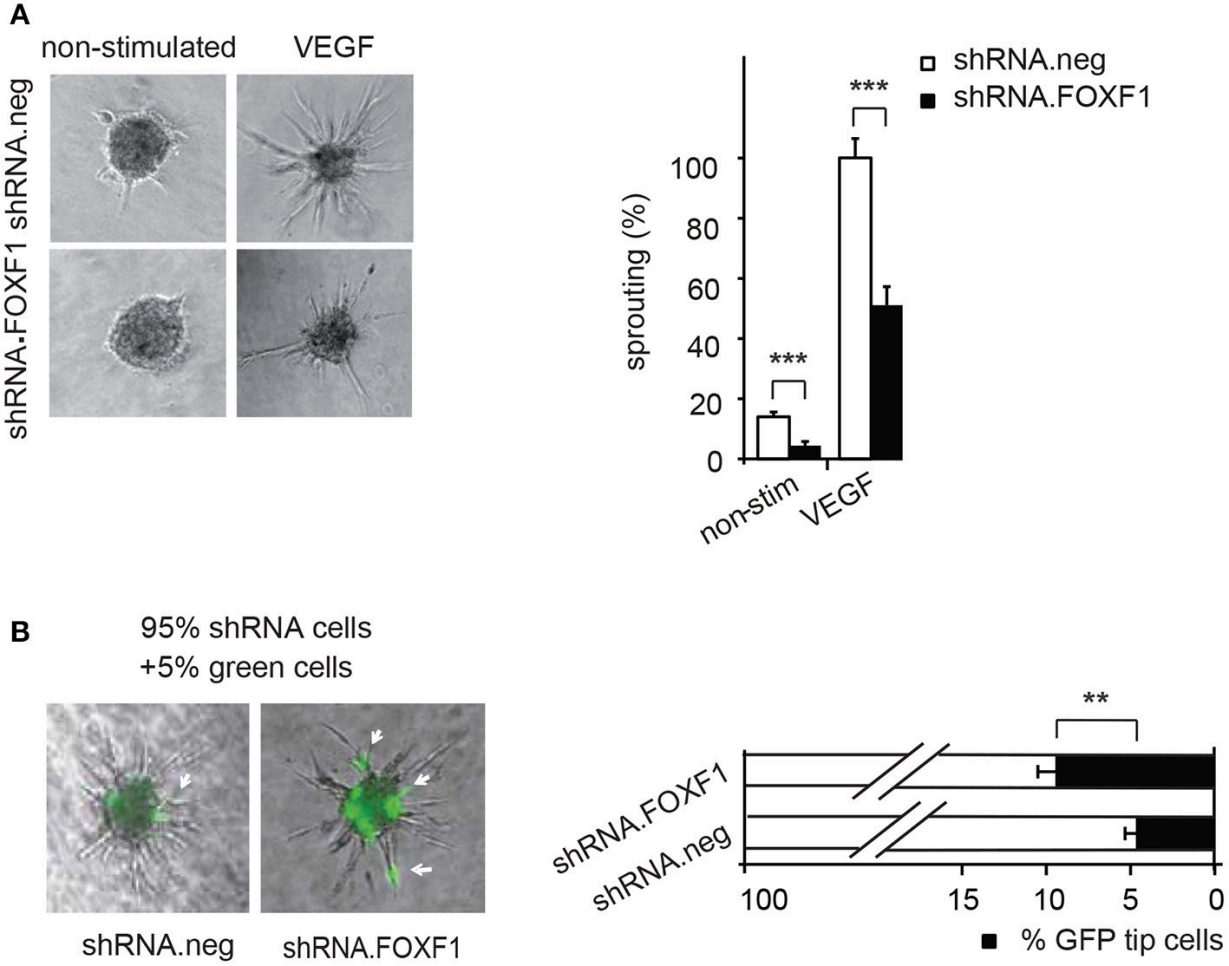
Downmodulation of FOXF1 reduces sprouting and the number of cells in tip cell position. **(A)** Downmodulation of FOXF1 reduces sprouting: Cells were transduced with lentiviruses expressing shRNA.FOXF1 or control viruses (shRNA.neg) for 48 h. Then the spheroid assay was performed without stimulation or in the presence of VEGF-A. Representative images of spheroids are displayed in the left panel. A corresponding quantification is depicted in the right panel and displayed as mean values ± SEM. The cumulative sprout lengths observed for spheroids transduced with shRNA.neg viruses and induced with VEGF-A were arbitrarily set to 100%. One representative experiment of 3 performed is shown. **(B)** Downmodulation of FOXF1 reduces the capacity of ECFC to generate tip cells: Cells were separately transduced with lentiviruses expressing shRNA.FOXF1 or control (shRNA.neg) lentiviruses for 48 h. Then the cells were mixed with 5% of cells transduced with a lentivirus encoding GFP and the spheroid assay performed. The number of GFP-expressing tip cells was scored. The left panel depicts representative pictures of spheroids comprised of combinations of 95% of shRNA-neg (left) or 95% of shRNA-FOXF1 (right) transduced cells mixed with 5% of GFP-expressing cells. Arrows indicate GFP expressing green cells in tip cell position. The right panel shows the quantification of green cells found in tip cell position. Mean percentages of green tip cells ± SEM calculated from 40 spheroids per sample and three experiments with different isolates of ECFC are shown (^**^p < 0.01; ^***^p < 0.001).

Vice versa, when we knocked down endogenous FOXF1 by shRNA-expressing lentiviruses a strongly reduced sprouting activity was observed in control as well as VEGF-A-induced assays (Figure 2A). These data are in line with an important role of FOXF1 for basal as well as VEGF-A-induced sprouting of ECFC.

Remarkably, it appeared from these analyses that especially the number of initiated sprouts per spheroid was strongly altered by FOXF1 and we therefore explored if FOXF1 influences the potential of ECFC to form tip cells. These are responsible for initiation of sprout formation, thereby controlling the number of sprouts. For this purpose, the influence of knocked-down FOXF1 expression on the probability of the cells to reach the tip cell position was evaluated in a mosaic expression experiment. ECFC infected with lentiviruses expressing shRNA-FOXF1 or control shRNA were mixed with 5% of ECFC infected with a lentivirus encoding GFP and then the spheroid sprouting assay was performed. Green tip cells with normal FOXF1 expression were scored in relation to non-GFP containing tip cells originating from cells transduced with either shRNA-FOXF1 or control shRNA. As expected from the mixing ratio, the combination of control shRNA transduced cells with 5% GFP expressing cells gave rise to 5% of green tip cells. However, when we co-cultured shRNA-FOXF1 expressing cells with 5% of GFP expressing cells it became apparent that downregulation of FOXF1 reduced the probability of these cells to reach tip cell position and nearly doubled the proportion of green tip cells with normal FOXF1 expression from 5 to about 10% (Figure 2B). This finding implies that FOXF1 controls sprouting via modulating the capacity of the cells to form tip cells and to initiate sprouting.

### FOXF1 specifically upregulates Notch2 receptor expression, which is involved in controlling sprouting activity

Given the impact of FOXF1 on sprouting, we analyzed the influence of the factor on the expression of surface proteins previously shown to determine the probability to form tip cells and to initiate sprouting. This included members of the Notch receptor family, since the Notch signaling pathway has been shown to be of crucial importance for tip cell generation (Phng and Gerhardt, 2009).

**Figure 3:**
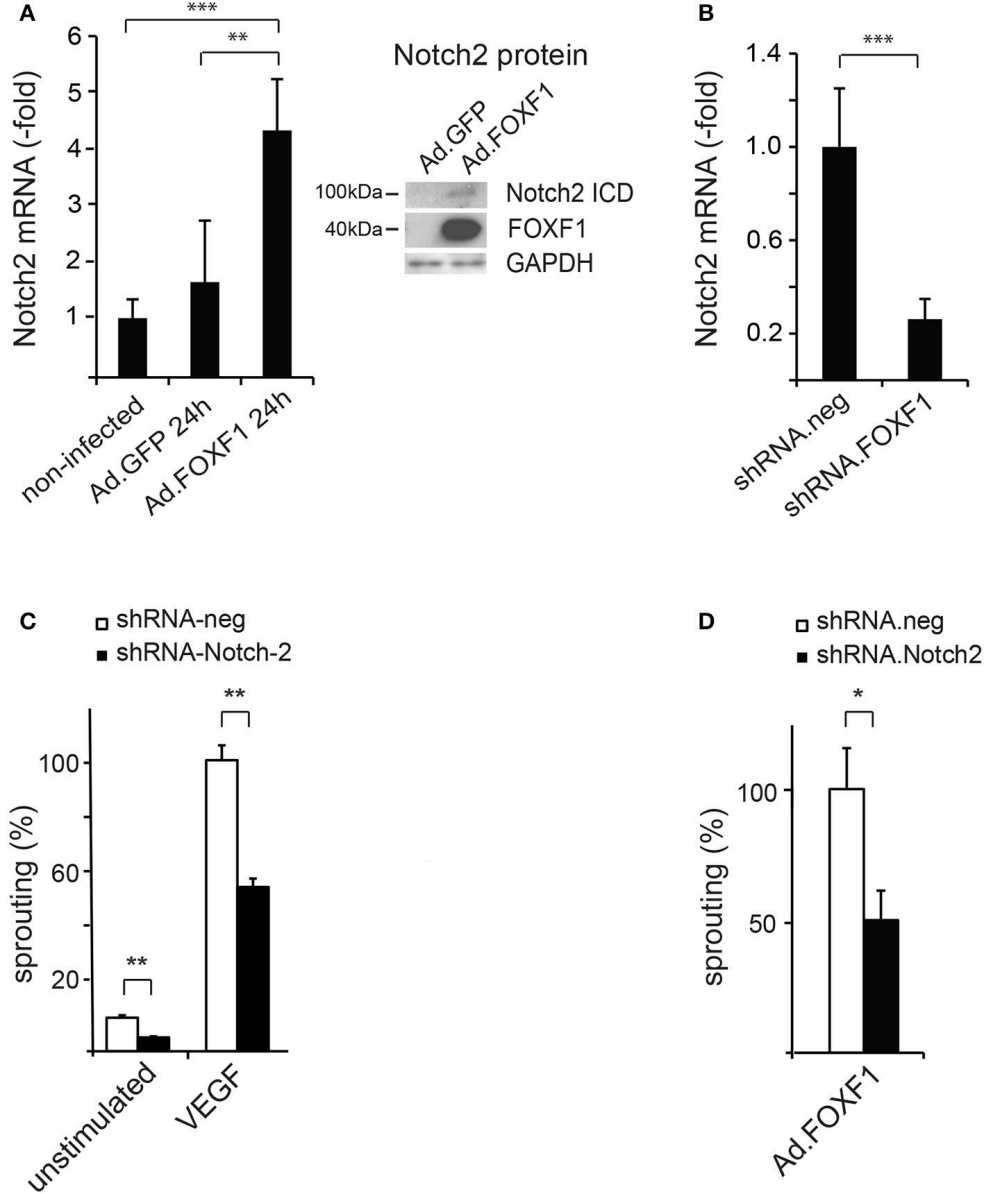
FOXF1 regulates Notch2 expression, which mediates sprouting capability. **(A)** FOXF1 overexpression upregulates Notch2 mRNA and protein: ECFC were transduced with Ad.FOXF1 or Ad.GFP for 24 h. The left panel displays the realtime RT-PCR analyse giving mean values ± SEM of one experiment representative of 4 performed. The right panel shows Western blot images obtained after incubation of membranes with antibodies recognizing the Notch2 intracellular domain or FOXF1. Blots were further reprobed with antibodies for GAPDH as expression controls. **(B)** Downmodulation of FOXF1 reduces Notch2 mRNA: Cells were transduced with lentiviruses expressing shRNA.FOXF1 or control viruses (shRNA.neg) for 48 h. Then total RNA was isolated and realtime RT-PCR analysis performed. Mean values ± SEM were calculated using ß-actin as an internal standard. **(C)** Downmodulation of Notch2 strongly reduces sprouting: ECFC were transduced with shRNA.Notch2 or shRNA.neg viruses for 48 h. Then the spheroid sprouting assay was performed without stimulation or using induction with VEGF-A. Results are displayed as mean values of the cumulative sprout length ± SEM. Results obtained with control virus-infected and VEGF-stimulated spheroids were arbitrarily set to 100%. One representative experiment of 3 performed is shown. **(D)** Downmodulation of Notch2 inhibits FOXF1-induced sprouting: ECFC were first transduced with shRNA.Notch-2 or shRNA.neg lentiviruses and after 24 h in addition infected with Ad.FOXF1. After another 24 h the spheroid sprouting assay was performed. Results are mean values ± SEM calculated from three experiments (^*^p < 0.05; ^**^p < 0.01; ^***^p < 0.001).

Indeed, when we quantified by realtime RT-PCR the expression of mRNAs for the major Notch receptors in response to FOXF1 overexpression we found Notch2 mRNA significantly upregulated. Western Blot analysis furthermore revealed an increase of an about 100 kD cleaved Notch2 fragment containing the intracellular domain of Notch2 that mediates Notch activity (Figure 3A). In line with a transcriptional upregulation of Notch2 by FOXF1, knocking down FOXF1 via short hairpin RNA caused a massive decrease of Notch2 mRNA (Figure 3B).

To investigate, if the effects of FOXF1 on sprouting were mediated via Notch2, we analyzed the potential influence of downmodulation of Notch2 on sprouting activity. A significant and selective shRNA-mediated downregulation of Notch2 was achieved by lentiviral transduction (Supplementary Figure S3). Intriguingly, this downmodulation resulted in a reduction of sprouting capacity (Figure 3C), which was similar to the reduced sprouting observed after knocking down FOXF1 (Figure 2A). Furthermore, we tested whether shRNA-mediated downmodulation of Notch-2 in Ad.FOXF1 transduced cells would reduce the FOXF1-induced sprouting. Indeed, a strong reduction of sprouting was obtained (Figure 3D). Taken together, these results provide strong evidence that the Notch2 receptor contributes to the initialization of sprout formation by FOXF1 in ECFC.

### FOXF1 promotes expression of ephrinb2 as well as VEGF receptor-2, whereas it reduces EphB4 expression

**Figure 4:**
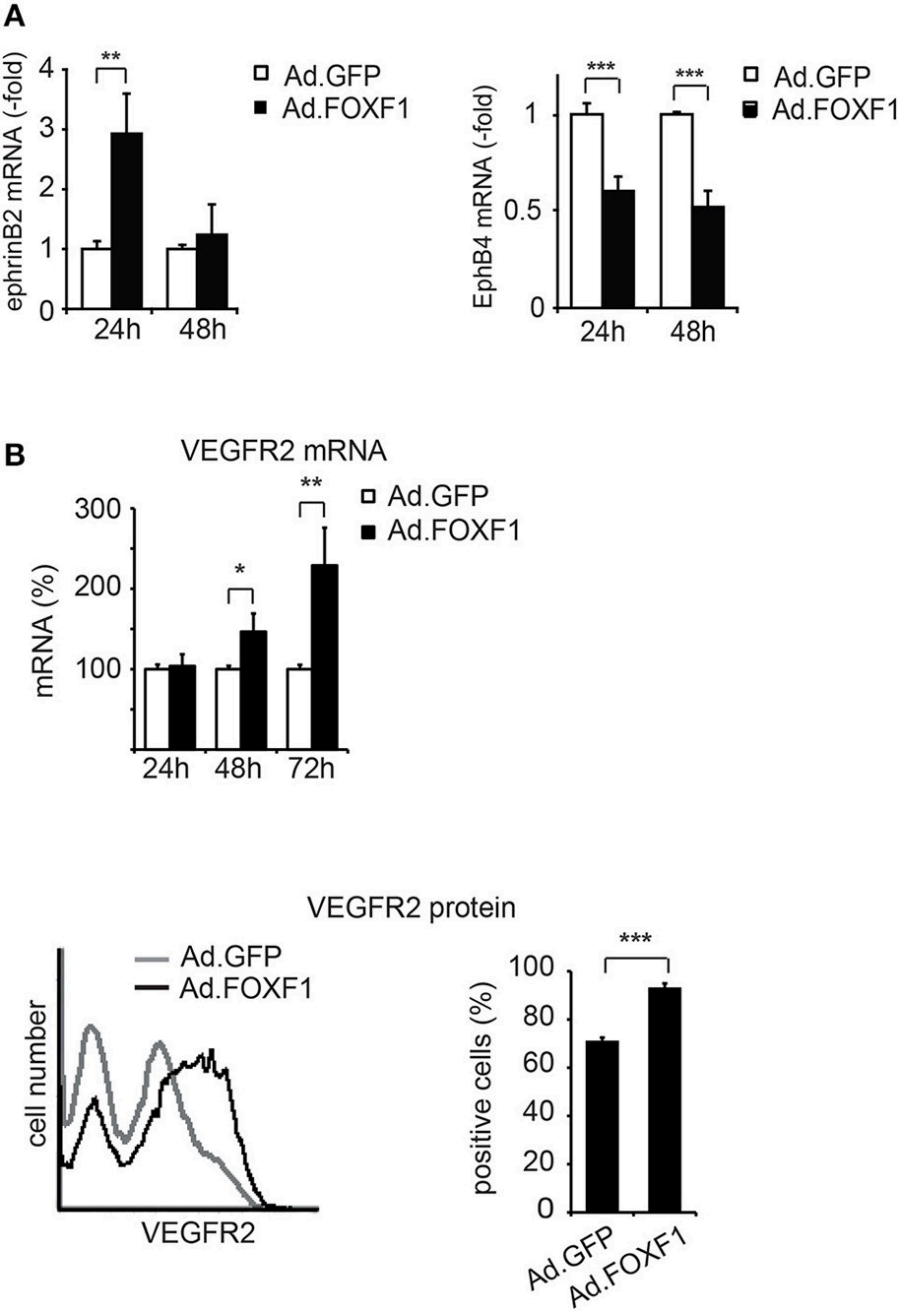
FOXF1 induces ephrinB2 and VEGF receptor-2, but reduces EphB4 expression. **(A)** FOXF1 overexpression upregulates ephrin B2 mRNA and downmodulates EphB4 mRNA: ECFC were infected with Ad.FOXF1 or control Ad.GFP for 24 and 48 h. Then total RNA was isolated and ephrinB2 (left panel) and EphB4 (right panel) mRNAs quantified by real-time RT-PCR analysis. Values were normalized to ß-actin mRNA as internal standard. Mean values ± SEM calculated from results obtained from three experiments with different donors are illustrated (^**^p < 0.01, ^***^p < 0.001). **(B)** FOXF1 overexpression upregulates expression of VEGFR-2 at mRNA and protein level: ECFCs were transduced for 24, 48, and 72 h. Then total RNA was isolated and real-time RT-PCR performed or cells were stained with anti-KDR antibodies and analyzed by flow cytometry. The upper panel depicts the real-time RT-PCR analysis, mean values ± SEM calculated from at least three different donors are depicted. Values were normalized to β-actin mRNA as internal standard. Asterisks label values with significant differences (^*^p < 0.01, ^**^p < 0.001). The lower left panel shows a representative flow cytometry histogram of cells 48 h after transduction of cells with Ad.FOXF1 (black line) compared to Ad.GFP infected cells (gray line). The lower right panel displays the quantification of the flow cytometry data, mean percentages ± SD of three independent experiments each performed in triplicates are depicted (^***^p < 0.0001).

Ephrin B2 has been shown to characterize arterial endothelial cells and to be upregulated in endothelial tip cells during sprouting (Sawamiphak et al., 2010; Salvucci and Tosato, 2012). We therefore tested whether FOXF1 could alter the expression of ephrinB2 and its counteracting receptor EphB4. Indeed, we found that not only the mRNA for the arterial marker ephrinB2 was significantly upregulated upon FOXF1 overexpression, but that concomitantly also the mRNA for the venous marker EphB4 was significantly downregulated (Figure 4A).

Furthermore, we have been interested to determine whether another regulator of sprouting activity, the VEGF receptor-2, is regulated by FOXF1. Indeed we find that VEGF receptor-2 is upregulated by FOXF1 at the mRNA and protein level (Figure 4B). By flow cytometry we detect a significant increase in surface expression of VEGFR-2, suggesting its contribution to the high sprouting activity of ECFC.

### FOXF1 contributes to the formation of intersegmental vessels as well as the lymphatic trunk

The zebrafish is an excellent model to investigate vessel formation and sprouting during embryogenesis as vasculo- and angiogenesis are highly stereotypical and tightly regulated by defined genetic programs, several transgenic zebrafish lines expressing fluorescent markers in the vascular system are highly established and they can be easily genetically modified (Hogan and Schulte-Merker, 2017).

**Figure 5:**
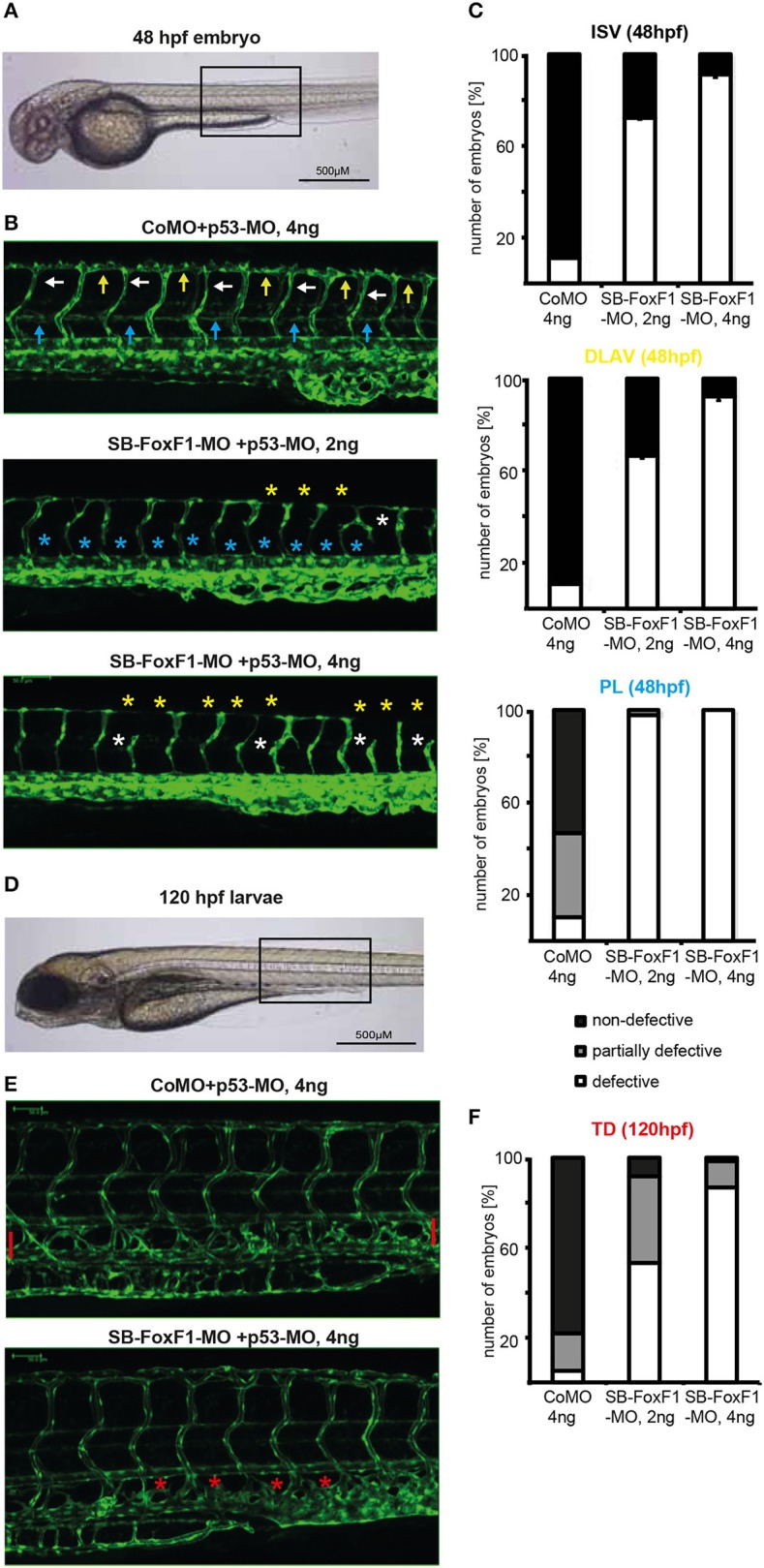
Downregulation of FOXF1 in zebrafish impairs vascular development. **(A–C)** Light and confocal microscopic images and quantification (at least 100 embryos per group) of tg(fli1:EGFP) embryos at 48 h post-fertilization injected with control (CoMO) or FoxF1 morpholino (SB-FoxF1-MO). Injection of the FoxF1 morpholino resulted in an impaired formation of the ISVs (white marks), DLAVs (yellow marks), and PLs (blue marks). **(D–F)** Light and confocal microscopic images and quantification (at least 100 embryos per group) of tg(fli1:EGFP) embryos at 120 h post-fertilization injected with control (CoMO) or FoxF1 morpholino (SB-FoxF1-MO). Injection of the FoxF1 morpholino resulted in an impaired formation of the TD (red marks).

To investigate a potential role of FOXF1 *in vivo* during embryonic vessel formation we downregulated zFOXF1 in zebrafish using a splice blocking morpholino (SB-FOXF1-MO) (Supplementary Figure S4). We observed at 48 hpf dose-dependent distorting effects on the formation of intersegmental vessels, which originate by sprouting from the dorsal aorta or posterior cardinal vein and usually regularly pervade every somite and eventually connect to the dorsal longitudinal anastomic vessel (Figures 5A–C). Aberrant numbers of intersegmental vessels, inefficient sprout formation and anastomosis of the dorsal longitudinal anastomosing vessel, or undirected sprouting patterns were observed. This was presumably at least in part due to defective sprouting of arterial vessels.

A second effect was the nearly entire absence of a forming lymphatic network presenting with nearly completely lacking parachordal lymphangioblasts at 48 hpf and a missing thoracic duct at 120 hpf (Figures 5D–F). These data suggest that in zebrafish FOXF1 plays an instructive role for arterial sprouting *in vivo*, and is additionally involved in organizing lymphatic vessel formation.

## Discussion

New blood vessels can form by two distinct mechanisms: first by vasculogenesis, a process in which in the embryo a vascular system is assembled de novo starting from endothelial progenitor cells; second by angiogenesis which enlarges the vasculature during growth by sprouting from existing vessels, endothelial proliferation and remodeling (Risau, 1997; Adams and Alitalo, 2007). Sprouting angiogenesis appears to be also the dominant form of neovascularization during wound repair and tumor formation (Sturtzel, 2017). The description of endothelial progenitor-like cells in circulating blood led to the concept of post-natal vasculogenesis, i.e., that EPC may contribute to angiogenic vessel growth and repair in the adult (Bautch, 2011). Whereas part of the cell types described in this context may be rather monocyte lineage-derived cells that contribute to angiogenesis by secretion of paracrine factors, a certain kind of human progenitors termed ECFC are believed to constitute true endothelial progenitors, to be descendants of progenitor cells resident in the vessel wall and to be competent to stimulate angiogenesis as well as to contribute to newly formed vessel wall endothelial cells (Banno and Yoder, 2017).

In this study we have been interested to identify characteristics of these progenitor cells of the vasculature with regard to transcription factors and surface receptors and their potential functional role. To gain evidence for specific functions defining the progenitor status of ECFC, we have comparatively investigated ECFC by transcriptomic profiling to vessel wall endothelial cells. We detected FOXF1, a member of the FOX family, as the most consistently overrepresented transcription factor in ECFC from human cord and adult blood. This is intriguing, as several FOX transcription factors have been described as pioneer transcription factors important for cell type-specific transcription defining different tissues (Bredenkamp et al., 2014; Iwafuchi-Doi et al., 2016; Braccioli et al., 2017). Furthermore, knock-down of FOXC2 in combination with Etv2 has been shown to disrupt vascular development in the zebrafish (De Val et al., 2008) and mouse embryo compound mutants for FOXC1 and FOXC2 display arteriovenous malformations (Seo et al., 2006). It was therefore possible that FOXF1 might control specific endothelial progenitor-type transcription and functions.

A hypothetical mechanism, how ECFC could contribute to neovascularization, is by invasion into hypoxic vessel areas followed by stimulation of angiogenic sprouting. This could be triggered via cell-to-cell contact inducing sprouting of neighboring endothelial cells, or by forming tip cells and initiating sprouts by the incorporated ECFCs themselves. That this is a conceivable process is supported by our finding that the sprouting capacities of ECFC are consistently higher than of vessel wall endothelial cells (Schneller and Hofer, unpublished observation).

We were therefore interested to see whether the high sprouting capacity of ECFC may be related to the high expression levels of FOXF1. Indeed, the level of FOXF1 expression determined the sprouting capacity of the cells as overexpression increased and knock-down strongly decreased sprouting. We also obtained evidence that the level of FOXF1 expression determined the probability of cells to reach tip cell position. These findings suggested that FOXF1 might control a transcriptional program that leads to the upregulation of proteins important for tip cell functions.

Among the surface molecules important for tip cell functions are members of the Notch family, VEGF receptors and ephrins (Adams and Alitalo, 2007; Adams and Eichmann, 2010). We were therefore first interested to test Notch family members. We detected that Notch2 was strongly upregulated on mRNA and protein level, whereas we could not detect differences for other Notch members. That Notch2 is directly regulated by FOXF1 was confirmed by downmodulation of FOXF1 resulting in diminished Notch2 expression. Importantly, knock-down of Notch2 further reduced *in vitro* angiogenic sprouting similar to knock-down of FOXF1. It also prevented increased sprouting induced by FOXF1 overexpression. This demonstrated that Notch2 is an important mediator of FOXF1 functions.

In this context it may be important that several lines of evidence suggest that Notch1 and Notch2 have different and at times opposing biological functions. For example, in a non-small cell lung cancer model, Notch2 mediates differentiation and has tumor suppressor function, whereas Notch1 promotes tumor initiation and progression (Baumgart et al., 2015). Similarly, Notch1 and Notch2 have been reported to have opposite prognostic effects in patients with colorectal cancer (Chu et al., 2011). Furthermore, in a study on mouse osteoclastogenesis Notch1 was found to inhibit, whereas Notch2 promoted the differentiation (Sekine et al., 2012). Although the details of the potential differential signaling and functions of Notch1 and Notch2 have not yet been clarified, it is conceivable that comparable mechanisms could lead to the increased sprouting capacity of ECFC with increased Notch2 expression, whereas Notch1 in stalk cells inhibits sprouting upon binding to Dll4 expressed on tip cells (Blanco and Gerhardt, 2013). Furthermore, there seem to be differences in the processing of the active intracellular domains as Notch2 cleavage appeared not to be sensitive to inhibition by standard concentrations of the γ-secretase inhibitor DAPT, which efficiently block Notch 1 cleavage (Fortini et al., 2017). A concurrent theme is the differential function of Notch ligands on sprouting. Whereas Dll4 on tip cells inhibits stalk cell sprouting via Notch, Jagged1 can activate sprouting by interfering with Dll4/Notch signaling (Benedito et al., 2009). In our work we have not further analyzed expression and contribution of Notch1 and of Notch ligands to sprouting of ECFC, the question of the ligand(s) interacting with Notch2 and a potential role of ligands such as Dll1 and Dll4 remains to be determined.

Additionally, while this work was already in progress, a study on FOXF1 knock-out in mice was published (Ren et al., 2014). It described that FOXF1 is required for formation of the embryonic vasculature and that this is in part due to effects on VEGF signaling. Using a Tie2-Cre-mediated conditional knock-out of the FOXF1 gene in endothelial cells, the authors found that FOXF1 deletion led to embryonic lethality between days 13.5 and 16.5, the embryos displaying growth retardation and abnormalities in various organs including cardiovascular defects. Furthermore, a decreased vascular branching in the yolk sac and placenta and a diminished number of blood vessels in the lung was observed. These defects were, as shown by chip analysis, to be likely caused in part by the direct regulation of angiogenesis related receptor genes by FOXF1, including VEGF receptor-1,−2, and Tie-2. In acccordance with this finding we show that FOXF1 overexpression upregulates surface expression of VEGF receptor-2. It is therefore likely that increased VEGF signaling via upregulation of VEGFR-2 contributes to the increased sprouting capabilities of ECFC.

Another surface protein implicated in important functions of tip cells as well as in arterial endothelial specification is ephrinB2 (Sawamiphak et al., 2010; Salvucci and Tosato, 2012). It has been shown that ephrinB2 reverse signaling via PDZ domain proteins regulates the guidance of endothelial tip cells and is important for efficient filopodial extensions at the vascular front. This is likely due to promoting of VEGFR-2 internalization and signaling. In addition, several studies have portrayed the key function of ephrinB2 in the determination of the arterial fate of endothelial cells, whereas its receptor EphB4 characterizes venous endothelial cells (Herbert et al., 2009).

When we tested a potential effect of FOXF1 on the expression of ephrinB2 and EphB4 we indeed detected an upregulation of ephrinB2 and a concomitant downmodulation of EphB4. These data suggest that the opposite effects of FOXF1 on ephrinB2 and EphB4 may favor arterial sprouting.

To substantiate a role of FOXF1 for *in vivo* sprouting we chose to investigate FOXF1 downmodulation by morpholino oligonucleotides (Blum et al., 2015) during zebrafish development as this model is an emerging disease model and allows to monitor consecutive cellular dynamics (Kirchberger et al., 2017). The first effect we observed was inefficient intersegmental vessel sprouting that starts from the dorsal artery about 1 day post fertilization. This led to flawed formation of the DLAV. This is in line with a preferential role of FOXF1 during arterial sprouting *in vivo*. Unexpectedly, a second phenotypic effect became apparent. Normally, during secondary venous angiogenesis and lymphangiogenesis, endothelial cells from secondary sprouts constitute a transient pool of lymphatic endothelial progenitors, called parachordal lymphangioblasts (PLs). These migrate individually and reassemble later to form the major trunk lymphatics, such as the thoracic duct (TD) (Hogan and Schulte-Merker, 2017). As shown in Figure 5, formation of the PLs at 48 hpf and TD at 120 hpf was strongly blocked. These data in zebrafish support a further role of FOXF1 during the generation, migration or reassembly of lymphatic progenitors. Whether this indicates a general role of FOXF1 for migrating endothelial progenitors that may contribute to post-natal vasculogenic processes will need further investigations.

In mammals, two lines of *in vivo* evidence further support a preferential role of FOXF1 in the development of lung capillaries. First, a conditional knock-out of FOXF1 in mice impairs the development of the pulmonary vascular plexus and leads to reduced numbers of capillaries in the lung (Ren et al., 2014). Second, in human patients alveolar capillary dysplasia (ACDMPV), a developmental disorder of the lung, has been shown to result from haploinsufficiency of FOXF1 (Stankiewicz et al., 2009; Sen et al., 2014). ACDMPV patients display drastically reduced numbers of capillaries and lobular underdevelopment. The importance of FOXF1 for correct lung vessel formation can be seen in line with the possibility that vasculogenic processes contribute to the development of lung vasculature (Peng et al., 2013; Gao et al., 2016) as well as with the hypothesis that lung capillaries could be a major origin of ECFC with preferential expression of FOXF1.

Taken together our data support that the forkhead box transcription factor FOXF1, aside being essential for embryonic vascular development, determines important properties of endothelial progenitor cells. These functions of FOXF1 appear to be mediated through the upregulation of angiogenesis-related genes that provide sprouting capabilities. We show here for the first time that Notch2 is a major mediator of FOXF1 functions, which emphasizes the importance of Notch signaling in the context of endothelial progenitors and suggests that a potential opposing interaction of different Notch isoforms needs to be considered for vascular sprouting and should be investigated in more detail. A further upregulation of ephrinB2 and of VEGF receptor-2, as seen by others (Ren et al., 2014) and also by us, contribute to the FOXF1-mediated sprouting capabilities. It remains to be determined to which extent those are linked to Notch signaling. Finally, it is tempting to speculate that based on these properties ECFC may function to initiate sprouting following integration into ischemic vessels.

In any case it is conceivable that FOXF1 could be used as a marker to identify proposed endothelial progenitor cells in the vessel wall. In addition, it is a candidate to engineer endothelial progenitor cells with improved sprouting capabilities for cell therapies of ischemic diseases or for the ex vivo generation of vascularized tissues.

## Author contributions

CS and KL did the majority of detailed planning, experimental work and analysis, as well as the first draft of the manuscript. JT, BE, JS, PQ, MB, AJ, and GU performed experiments, analyzed data, and generated individual figures. K-HP, EG, RdM, and RH-W contributed to the design of the study and to conclusions from the results. JK designed and interpreted the zebrafish experiments. EH perceived and designed the research project and corrected and finished the manuscript.

### Conflict of interest statement

AJ and K-HP were employed by the company VivoCell Biosolutions GmbH, Graz, Austria. The remaining authors declare that the research was conducted in the absence of any commercial or financial relationships that could be construed as a potential conflict of interest.
