# Supplementary material for: FOXF1 Mediates Endothelial Progenitor Functions and Regulates Vascular Sprouting
Source: Front Bioeng Biotechnol. 2018 Jun 14;6:76. doi: 10.3389/fbioe.2018.00076 (PMC6010557; doi:10.3389/fbioe.2018.00076)
Supplement: Supplementary file 1 [file Table_1.PDF]

Supplementary Material

FOXF1 Mediates Endothelial Progenitor Functions and Regulates Vascular Sprouting

Caterina Sturtzel, Karoline Lipnik, Renate Hofer-Warbinek, Julia Testori, Bettina Ebner, Jacqueline Seigner, Ping Qiu, Martin Bilban, Anita Jandrositz, Karl-Heinz Preisegger, Gerold Untergasser, Eberhard Gunsilius, Rainer de Martin, Jens Kroll and Erhard Hofer*

*** Correspondence:**
Erhard Hofer
erhard.hofer@meduniwien.ac.at
erhard.hofer@gmx.at

LEGENDS TO SUPPLEMENTARY TABLES

Supplementary Table S1. Comparison of gene expression levels between ECFC, vessel wall endothelial cells and hematopoietic CD34$^+$ cells.

ECFC were obtained from cord or adult blood and HUVEC or HSVEC from umbilical cords or saphenous veins, respectively. CD34$^+$ cells were obtained by magnetic sorting from cord blood. Freshly isolated CD34$^+$ cells and second passage ECFC and endothelial cells were used for RNA isolation. Then Affymetrix microarray analysis was performed.

(A) List of the five genes most differentially expressed between ECFC and vessel wall endothelial cells.

Fold differences in the expression levels of genes are given for cord blood ECFC versus HUVEC (upper part) and adult blood ECFC versus HSVEC (lower part). Genes are listed according to decreasing differences.

(B) Comparison of gene expression levels between cord blood ECFC, HUVEC and cord blood CD34$^+$ cells.

Fold differences in the expression levels of genes are displayed for ECFC versus HUVEC (left column) and ECFC versus CD34$^+$ cells (right column) for two transcription factors found to be preferentially expressed in ECFC as well as selected progenitor, endothelial and hematopoietic/monocytic markers.

Supplementary Table S2. List of primers used for realtime RT-PCR.

UniGene ID	Entrez gene	Gene Symbol	Gene title	cord blood ECFC/ HUVEC
Hs.155591	2294	FOXF1	forkhead box F1	19,98
Hs.591836	79618	HMBOX1	homeobox containing 1	6,86
Hs.33446	2494	NR5A2	nuclear receptor subfamily 5, group A, member 2	5,98
Hs.250693	51351	ZNF117	zinc finger protein 117	5,37
Hs.697304	3090	HIC1	hypermethylated in cancer 1	4,81

UniGene ID	Entrez gene	Gene Symbol	Gene title	peripheral blood ECFC/ SVEC
Hs.155591	2294	FOXF1	forkhead box F1	10,61
Hs.510989	4212	MEIS2	Meis homeobox 2	7,56
Hs.250693	51351	ZNF117	zinc finger protein 117	6,01
Hs.125962	22797	TFEC	transcription factor EC	5,64
Hs.41683	8092	ALX1	ALX homeobox 1	5,48

Supplementary Table S1A.

UniGene ID	Entrez Gene	Gene Symbol	Gene Title	ECFC / HUVEC	ECFC / CD34+
**Transcription factors with preferential expression in ECFC from cord blood and adult blood**					
Hs.155591	2294	FOXF1	forkhead box F1	19,98	24,25
Hs.250693	51351	ZNF117	zinc finger protein 117	5,37	9,37
**Progenitor markers**					
Hs.374990	947	CD34	CD34 molecule	1,82	0,26
Hs.614734	8842	PROM1 (CD133)	prominin 1	0,83	0,0024
**Vascular markers**					
Hs.479756	3791	KDR	vascular endothelial growth factor receptor-2	2,92	58,23
Hs.654360	2321	FLT1	vascular endothelial growth factor receptor-1	0,52	8,95
Hs.89640	7010	TEK (Tie2)	TEK tyrosine kinase, endothelial	0,60	8,09
Hs.76206	1003	CDH5	cadherin 5, type 2, VE-cadherin (vascular epithelium)	1,25	88,21
Hs.440848	7450	VWF	von Willebrand factor	0,28	6,74
Hs.514412	5175	PECAM1 (CD31)	platelet/endothelial cell adhesion molecule (CD31)	0,81	4,39
**Hematopoietic/monocytic markers**					
Hs.654514	5788	PTPRC (CD45)	protein tyrosine phosphatase, receptor type, C	1,12	0,0074
Hs.163867	929	CD14	CD14 molecule	0,80	0,031

Supplementary Table S1B

Primer name	Sequence
notch-2 fw	AACACGGTCGAGTGCCTGTT
notch-2 rev	CCTGGTCACAGTGGTTGTCT
FOXF1 fw	CGTATCTGCACCAGAACAGC
FOXF1 rev	ACTACCACCAGCAGGTCACC
Ephrin B2 fw	CTGCTGGATCAACCAGGAAT
Ephrin B2 rev	GATGTTGTTCCCCGAATGTC
EphB4 fw	GGACCAGCGGCAGCCTCACTA
EphB4 rev	CGGAGCAGGTCCTCAGCAGAGATCT
VEGFR-2 fw	ACTTTGGAAGACAGAACCAAATTATCT
VEGFR-2 rev	TGGGCACCATTCCACCA

Supplementary Table S2

LEGENDS TO SUPPLEMENTARY FIGURES

Supplementary Figure S1. Immunocytochemical staining of endothelial markers on ECFC.

ECFC were grown to density on tissue chamber slides and stained with FITC-labeled antibodies for CD31 (left), VE-cadherin (middle) and von Willebrand factor (vWF, right). Microscopic images were taken at 400x magnification.

Supplementary Figure S2. Overexpression and downmodulation of FOXF1.

(A) Overexpression of FOXF1 by recombinant adenoviruses. ECFC were transduced with control adenovirus (Ad.GFP) or with FOXF1 expressing adenoviruses (Ad.FOXF1) for 48 h using a MOI of 8. Then RNA was isolated and realtime RT-PCR performed (left panel) or total cell lysates were prepared and proteins separated by SDS-polyacrylamide gel electrophoresis, Western blotted and the membranes probed with anti-FOXF1 antibody (right panel). Results for FOXF1 mRNA levels are displayed after normalization to b-actin as fold of the levels obtained for ECFC transduced with Ad.GFP +/- SD calculated from triplicate wells. FOXF1 protein levels are displayed on a shortly exposed Western blot for Ad.GFP and AD.FOXF1 cultures, endogenous levels of FOXF1 protein in non-transduced ECFC are displayed on a long exposure on the right. One exemplary experiment from three performed is shown.

(B) Downmodulation of FOXF1 by shRNA expressing lentiviruses. ECFC were transduced with control lentiviruses containing a scrambled shRNA (shRNA.control) or lentiviruses encoding shRNA for FOXF1 (shRNA.FOXF1). 48 h after transduction RNA was isolated for realtime RT-PCR and cell lysates obtained for Western blots. Results for FOXF1 mRNA levels are displayed after normalization to b-actin as fold of the levels obtained for ECFC transduced with control shRNA +/- SD calculated from triplicate wells (left panel). Downmodulation of FOXF1 protein and constant levels of GAPDH is shown on the Western blot (right panel). One exemplary experiment from three performed is shown.

Supplementary Figure S3. Downmodulation of Notch2.

ECFC were transduced with lentiviruses expressing shRNA for Notch2 (shRNA.Notch2) or control lentiviruses with a scrambled shRNA (shRNA.control). After 48 h RNA was isolated and realtime RT-PCR performed to detect Notch2 mRNA levels (left panel). Results for Notch2 mRNA levels are displayed after normalization to b-actin as fold of the levels obtained for ECFC transduced with control shRNA +/- SD calculated from triplicate wells. In addition, levels of Notch1, Notch2 and Notch3 were tested by semi-quantitative RT-PCR followed by separation of the specific obtained PCR products on an agarose gel and staining with ethidium bromide (right panel).

Supplementary Figure S4. Expression silencing of zFOXF1 in zebrafish

Expression silencing as shown by RT-PCR of zFOXF1 in 24hpf zebrafish embryos using 2ng or 4ng of a splicing-blocking morpholino SB-FoxF1-MO+p53 MO. The upper signal represents the wildtype splice product, the lower signal the morphant splice products. Injection of the morpholino generated a substantial loss of the wildtype splice product.

ECFC

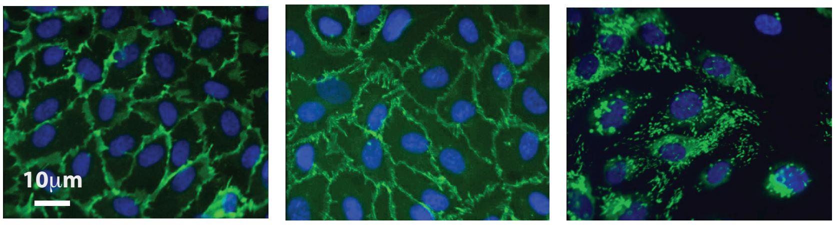

CD31 VE-cadherin vWF

Suppl. Figure 1

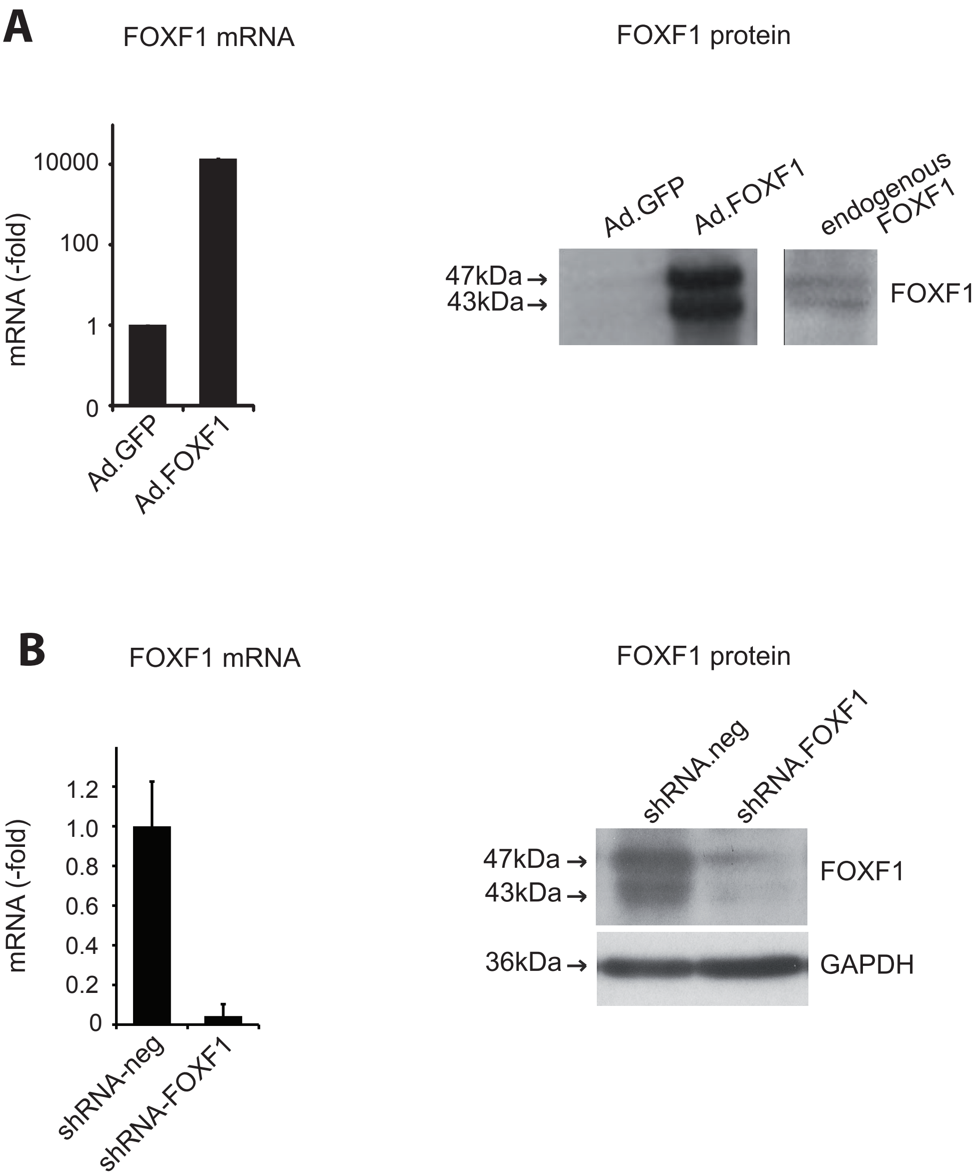

Suppl. Figure 2

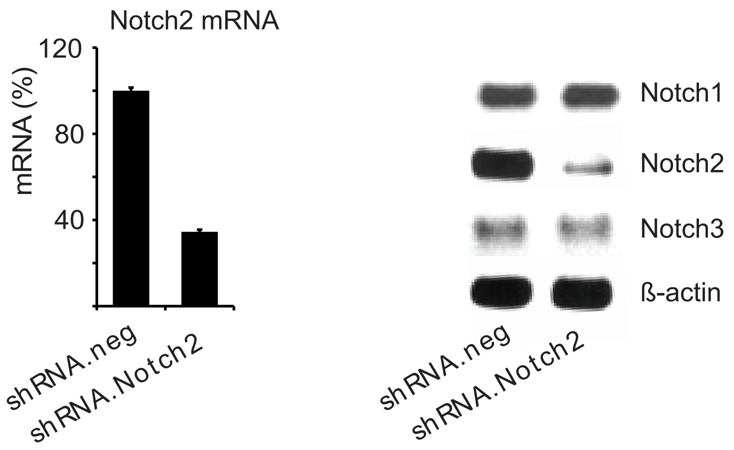

Suppl. Figure 3

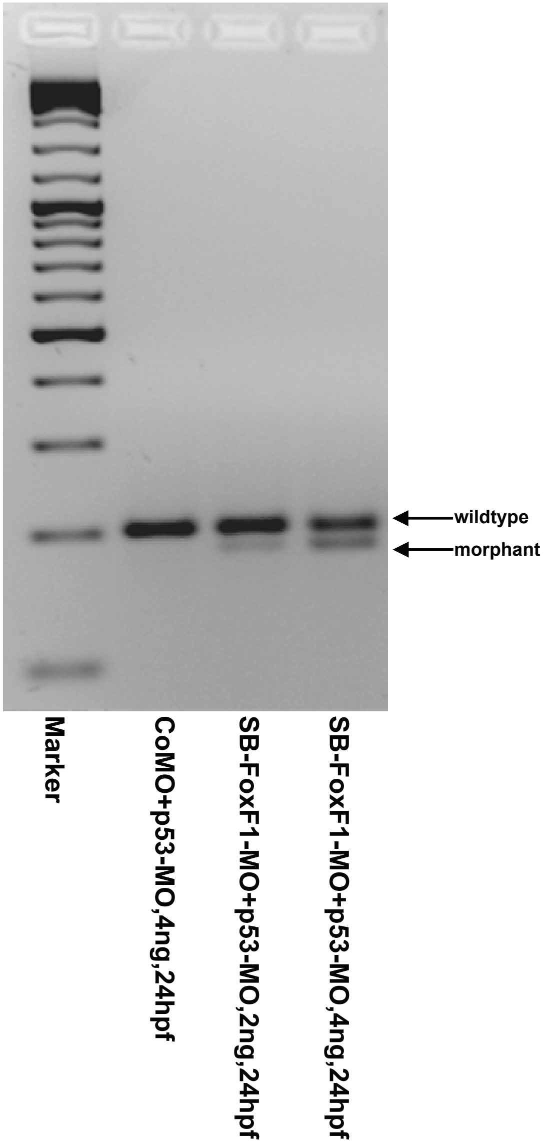

Suppl. Figure 4
